# Supplementary material for: VAP spatially stabilizes dendritic mitochondria to locally support synaptic plasticity
Source: Nat Commun. 2024 Jan 4;15:205. doi: 10.1038/s41467-023-44233-8 (PMC10766606; doi:10.1038/s41467-023-44233-8)
Supplement: Supplementary file 1 — Supplementary Information [file 41467_2023_44233_MOESM1_ESM.pdf]

VAP spatially stabilizes dendritic mitochondria to locally support synaptic plasticity

Supplementary Information

Supplementary Figures

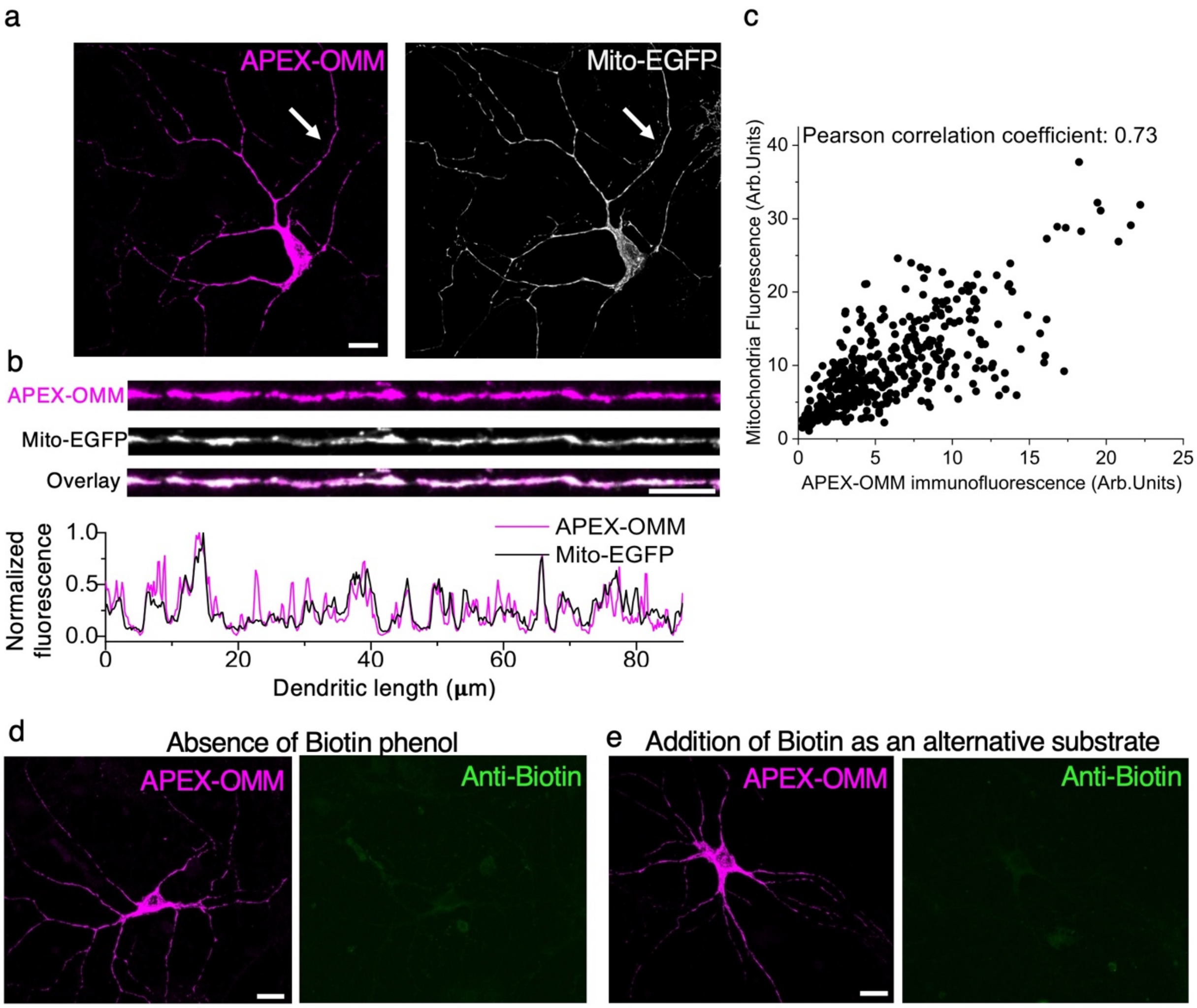

Figure 1. Control experiments related to APEX-OMM expression and biotin labeling. a APEX-OMM (magenta, same as in Figure **1b**) and mitochondria fluorescence (white, Mito-EGFP) from the same neuron overlap. Scale bar, 20 μm. **b** Line profiles of the dendrites pointed in **a** (white arrows) show an overlap between APEX-OMM and mitochondria fluorescence. Scale bar 10 μm. **c** Representative correlation between individual fluorescent pixel intensities of APEX-OMM and mitochondria in **a** shows a strong correlation. total n: 9 neurons, 2 animals. Representative neuron images expressing APEX-OMM (magenta), showing the absence of biotin labeling (green) in the absence of biotin phenol (**d**) or the presence of biotin as an alternative

substrate (**e**). total n in neurons, animals: 19, 5 (**d**), 14, 3 (**e**). Scale bar, 10 μm. Source Data files are provided.

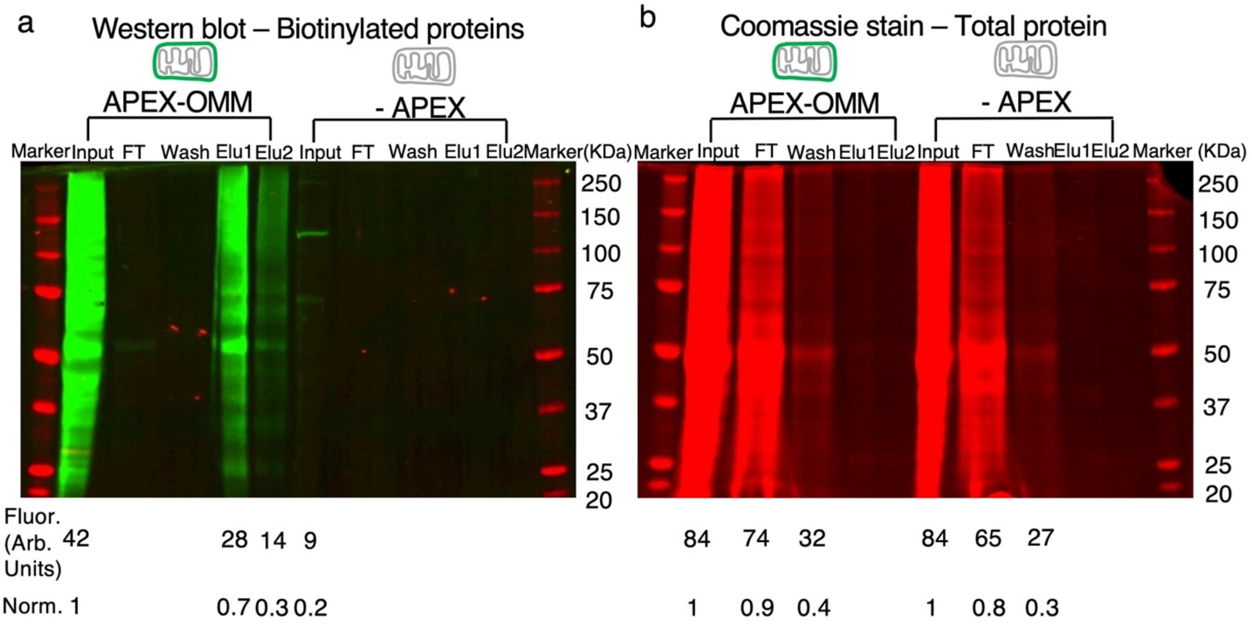

Figure 2. Streptavidin enrichment of the biotin-labeled APEX-OMM proteome. a Western blot analysis of the biotin-labeled, streptavidin-enriched proteome using anti-biotin immunodetection show significant biotin labeling in the input (Input) and eluate 1 (Elu1) of APEX-OMM samples, but not in -APEX (Control) samples. The residual biotin signal in -APEX (Control) represents endogenous biotinylated proteins present in mammalian cells[1]. **b** Coomassie staining analysis of the same experiment shows equal amounts of protein input used for APEX-OMM and -APEX (Control) samples, and most non-biotinylated proteins were washed out in the flow through (FT). Inset, Raw fluorescence intensities (Fluor.) and fluorescence normalized to APEX-OMM input (Norm.) for the respective lanes. Total n: 3 biological replicates, 3 animals.

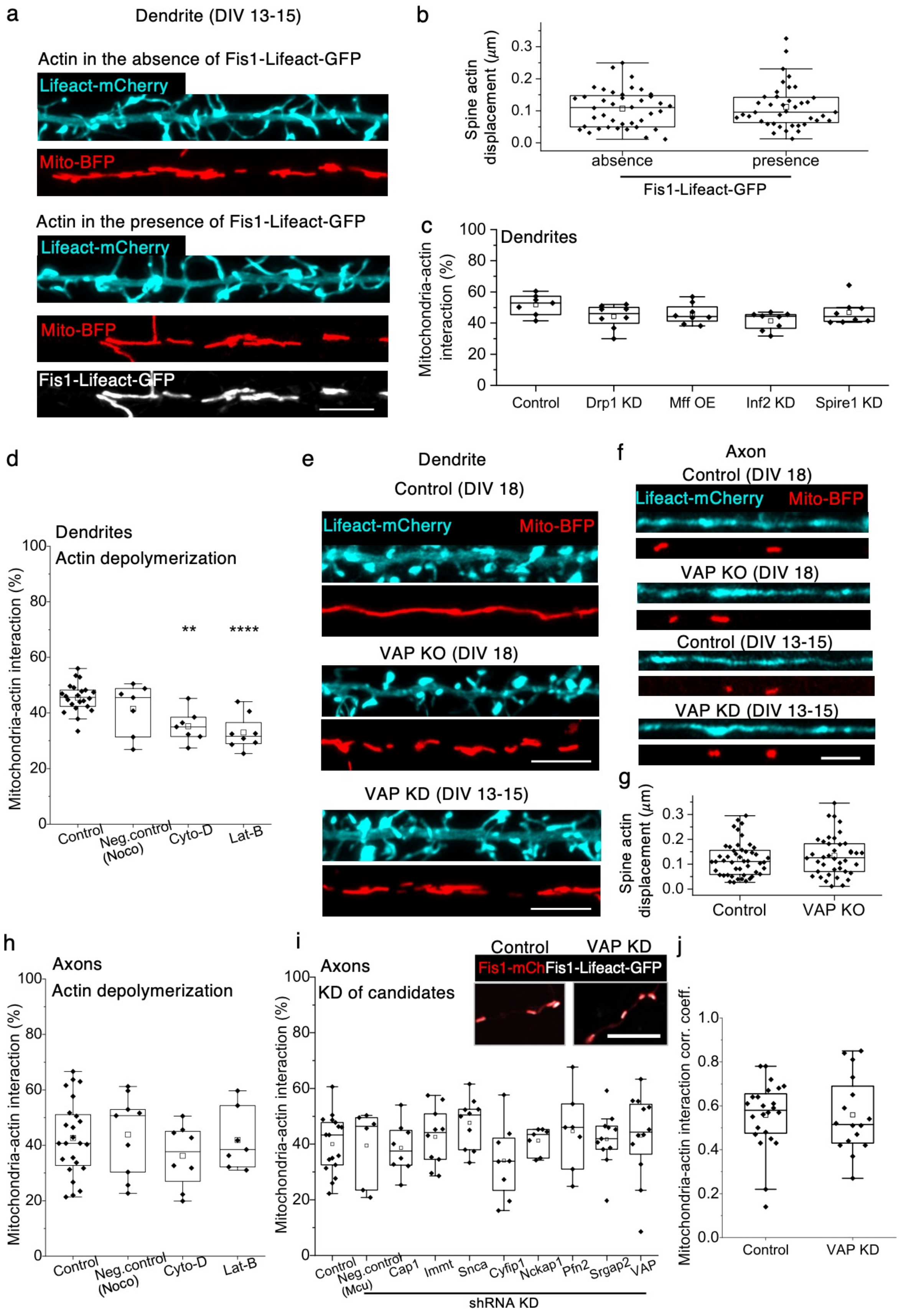

a
Dendrite (DIV 13-15)
Actin in the absence of Fis1-Lifeact-GFP
Lifeact-mCherry
Mito-BFP
Actin in the presence of Fis1-Lifeact-GFP
Lifeact-mCherry
Mito-BFP
Fis1-Lifeact-GFP
b
Spine actin displacement (μm)
absence
presence
Fis1-Lifeact-GFP
c
Dendrites
Mitochondria-actin interaction (%)
Control
Drp1 KD
Mff OE
Inf2 KD
Spire1 KD
d
Dendrites
Actin depolymerization
Mitochondria-actin interaction (%)
Control
Neg.control (Noco)
Cyto-D
Lat-B
e
Dendrite
Control (DIV 18)
Lifeact-mCherry
Mito-BFP
VAP KO (DIV 18)
VAP KD (DIV 13-15)
f
Axon
Control (DIV 18)
Lifeact-mCherry
Mito-BFP
VAP KO (DIV 18)
Control (DIV 13-15)
VAP KD (DIV 13-15)
g
Spine actin displacement (μm)
Control
VAP KO
h
Axons
Actin depolymerization
Mitochondria-actin interaction (%)
Control
Neg.control (Noco)
Cyto-D
Lat-B
i
Axons
KD of candidates
Control
VAP KD
Fis1-mCh
Fis1-Lifeact-GFP
Mitochondria-actin interaction (%)
Control
Neg.control (Mcu)
Cap1
Immt
Snca
Cyfip1
Nckap1
Pfn2
Srgap2
VAP
shRNA KD
j
Mitochondria-actin interaction corr. coeff.
Control
VAP KD

Figure 3. Control experiments for Mitochondrial-actin interaction measurements. a Representative Airyscan images (of **b**) of dendrites expressing Lifeact-mCherry (cyan), Mito-BFP (red), and Fis1-Lifeact-GFP (white). Scale bar, 5 μm. **b** Average spine actin displacement on Fis-Lifeact-GFP expression. n in spines, animals: 40, 1 (+/- Fis1-Lifeact-GFP). Two sample t-Test, p: 0.7. **c** Average mitochondria-actin interaction on lengthening (Drp KD), shortening mitochondria (Mff OE), or knocking down the mitochondrial fission factors Inf2 and Spire1. n in dendrites, animals: 7, 4 (Control), 8, 2 (Drp1 KD), 8, 3 (Mff OE), 8, 2 (Inf2 KD), 8, 2 (Spire1 KD). One-way ANOVA, Tukey test, p: 0.25 (Drp1 KD), 0.48 (Mff OE), 0.052 (Inf2 KD), 0.65 (Spire1 KD). **d** Average mitochondria-actin interaction on actin depolymerization using Cytochalasin-D (Cyto-D) and Latrunculin-B (Lat-B), and microtubule depolymerization using Nocodazole (Noco). n in dendrites, animals: 22, 6 (Control), 7, 2 (Noco), 7, 2 (Cyto-D), 8, 2 (Lat-B). One-way ANOVA, Tukey test, p: 0.45 (Noco), 0.002 (Cyto-D), 8.8 X 10^{-5} (Lat-B). Representative Airyscan images (of **g**) of dendrites (**e**) and axons (**f**) expressing Lifeact-mCherry (cyan) and Mito-BFP (red). n in neurons, animals: 3, 1 (Control), 7, 2 (VAP KD). Scale bar, 5 μm for **e** and 2 μm for **f**. **g** Average spine actin displacement. n in spines, animals: 48, 1 (Control), 40, 1 (VAP KO). Two sample t-Test, p: 0.4. Average mitochondria-actin interaction on actin and microtubule depolymerization (**h**, n in axons, animals: 24, 6 (Control), 9, 2 (Noco), 8, 2 (Cyto-D), 7, 2 (Lat-B), One-way ANOVA, Tukey test, p: 0.6) and on knocking down the 8 proteins. (**i**, n: 17, 9 (Control), 6, 2 (Mcu), 8, 2 (Cap1), 10, 4 (Immt), 10, 2 (Snca), 8, 2 (Cyfip1), 7, 2 (Nckap1), 7, 2 (Pfn2), 11, 5 (Srgap2), 12, 2 (VAP). One-way ANOVA, Tukey test, p: 0.5). (**i**, inset) Representative Airyscan images of **i** showing mitochondrial (red) interaction with actin (white). Scale bar, 5 μm. **j** Average correlation coefficient between mitochondria (Fis1-mCherry) and actin (Fis-Lifeact-GFP) in cell bodies in Control and VAP KD. n in regions, cell bodies, animals: 24, 4, 1 (Control), 16, 3, 1 (VAP KD). Two sample t-Test, p: 0.98. Source Data files are provided.

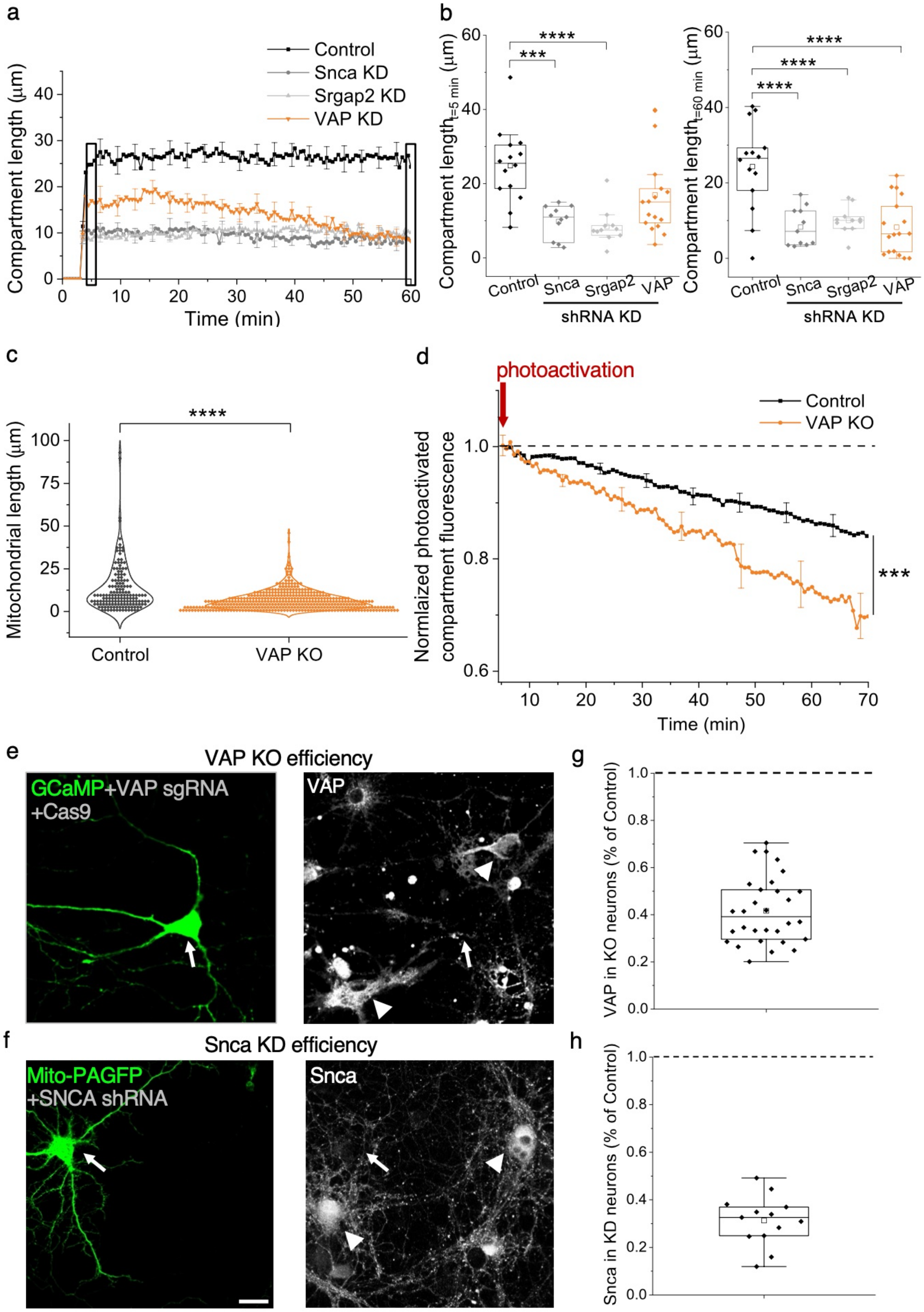
a
Control
Snca KD
Srgap2 KD
VAP KD
Compartment length (μm)
Time (min)
b
Compartment length$_{t=5\ min}$ (μm)
Compartment length$_{t=60\ min}$ (μm)
Control
Snca
Srgap2
VAP
shRNA KD
c
Mitochondrial length (μm)
Control
VAP KO
d
photoactivation
Control
VAP KO
Normlaized photoactivated compartment fluorescence
Time (min)
e
VAP KO efficiency
GCaMP+VAP sgRNA +Cas9
VAP
g
VAP in KO neurons (% of Control)
f
Snca KD efficiency
Mito-PAGFP +SNCA shRNA
Snca
h
Snca in KD neurons (% of Control)

Figure 4. Determination of mitochondrial compartment length and gene deletion efficiency. **a** Average time course of mitochondrial compartment lengths in Control (black), Snca KD (gray), Srgap2 KD (light gray), and VAP KD (orange). n in dendrites, animals: 14, 2 (Control), 11, 2, (Snca KD), 11, 2, (Srgap2 KD), 17, 4, (VAP KD). **b** Average compartment lengths from **a** (black rectangles) measured at 5 and 60 min post-photoactivation in Snca, Srgap2, and VAP KD at 60 minutes post-photoactivation. n: same as in **a**. Compartment $\text{length}_{t=5\ min}$: 24.8 $\pm$ 2.7 μm (Control), 9.9 $\pm$ 1.3 μm (Snca KD), 8.4 $\pm$ 1.4 μm (Srgap2 KD), 17.0 $\pm$ 2.7 μm (VAP KD); Compartment $\text{length}_{t=60\ min}$: 24.3 $\pm$ 3.1 μm (Control), 8.3 $\pm$ 1.5 μm (Snca KD), 10.1 $\pm$ 1.1 μm (Srgap2 KD), 8.2 $\pm$ 1.8 μm (VAP KD). One-way ANOVA, Tukey test, p for 5, 60 min: 5.65 X 10^{-4}, 3.79 X 10^{-5} (Snca KD), 1.53 X 10^{-4}, 2.45 X 10^{-4} (Srgap2 KD), 0.08, 4.4 X 10^{-6} (VAP KD). **c** Average mitochondrial length in Control and VAP KO. n in mitochondria, animals: 156, 2 (Control), 395, 2 (VAP KO). Two sample t-Test, p: 1.69 X 10^{-14}. **d** Average time course of photoactivated mitochondrial compartment shows a modest decrease in Control (black) and a statistically significant decrease in VAP KO (orange). The red arrow denotes the photoactivation time point. n in dendrites, animals: 14, 2 (Control), 11, 2 (VAP KO). Two sample t-Test, p: 8.28 X 10^{-4}. Representative images (of **g**, **h**) of transfected neurons (white arrow) expressing GCaMP or Mito-PAGFP (green) and VAP CRISPR-Cas9 sgRNAs (**e**) or Snca shRNA (**f**), and immunostained for VAP (**e**, white) or Snca (**f**, white) show reduced VAP and Snca immunofluorescence compared to untransfected, Control neurons (white arrowheads), identified using Map2. Similar results were obtained with VAP shRNA KD. Scale bar, 20 μm. Reduced average VAP (**g**) and Snca (**h**) immunofluorescence in VAP KO and Snca KD neurons, respectively, compared to untransfected, Control neurons. n in neurons, animals: 30, 10 (VAP KO), 13, 2 (Snca KD). Due to lack of reliable antibodies for Srgap2, successful expression of Srgap2 shRNA was confirmed with Td-Tomato (Fig. **4a**). Source Data files are provided.

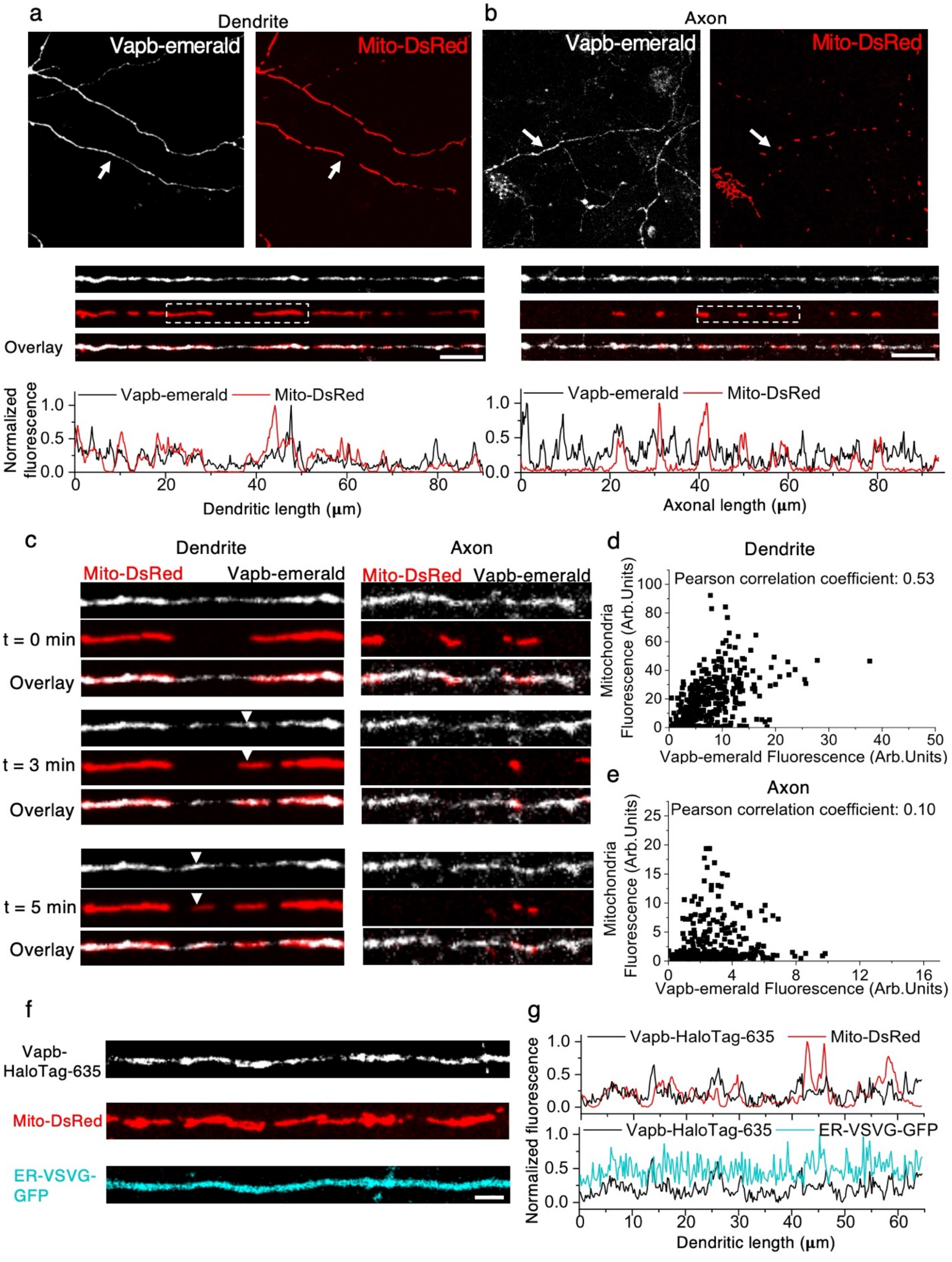
a
Dendrite
Vapb-emerald
Mito-DsRed
Overlay
Normalized fluorescence
Vapb-emerald
Mito-DsRed
Dendritic length (μm)
b
Axon
Vapb-emerald
Mito-DsRed
Axonal length (μm)
c
Dendrite
Axon
Mito-DsRed
Vapb-emerald
t = 0 min
t = 3 min
t = 5 min
Overlay
d
Dendrite
Pearson correlation coefficient: 0.53
Mitochondria Fluorescence (Arb.Units)
Vapb-emerald Fluorescence (Arb.Units)
e
Axon
Pearson correlation coefficient: 0.10
f
Vapb-HaloTag-635
Mito-DsRed
ER-VSVG-GFP
g
Vapb-HaloTag-635
Mito-DsRed
ER-VSVG-GFP
Normalized fluorescence
Dendritic length (μm)

Figure 5. Vapb is enriched near dendritic but not axonal mitochondria in live neurons. a Representative images (of Fig. **5i**) of dendrites from live neurons expressing Vapb (Vapb-emerald, white) and mitochondria (Mito-DsRed, red). White arrows depict dendrites straightened and magnified below for better visualization and line profiles showing enrichment of Vapb near dendritic mitochondria in live neurons (inset). Scale bar, 5 μm. **b** Representative images of axons from live neurons expressing Vapb (Vapb-emerald, white) and mitochondria (Mito-DsRed, red). White arrows depict axons straightened and magnified below for better visualization and line profiles showing negligible enrichment of Vapb near axonal mitochondria in live neurons (inset). As the Vapb signal is too low in axons, the brightness/contrast was adjusted for better visualization, making the background noise more visible in the axonal image compared to the dendrite image in **a**. Scale bar, 5 μm. **c** Representative timelapse images of the dendritic and axonal segments from the insets in **a** and **b** (white dotted box) at t = 0, 3, and 5 min. In dendrites, when mitochondria undergo fission and move away from the parent mitochondria (Mito-Dsred, red, white arrowheads), they remain enriched with Vapb (Vapb-emerald, white, white arrowheads). However, Vapb enrichment was not seen in moving axonal mitochondria. Representative correlation (of **5i**) between individual fluorescent pixel intensities of mitochondria (Mito-DsRed) and Vapb (Vapb-emerald) show a strong correlation in the dendrite (**d**) but not in the axon (**e**). Summarized data is available in Figure 5i. **f** Representative images of dendrites from live neurons expressing Vapb (Vapb-HaloTag-635, white), mitochondria (Mito-DsRed, red), and endoplasmic reticulum (ER-VSVG-GFP, cyan). Scale bar, 5 μm. **g** Line profile showing that Vapb is enriched near dendritic mitochondria but is not distributed throughout the ER at confocal resolution. Total n: 3 neurons, 1 animal. Source Data files are provided.

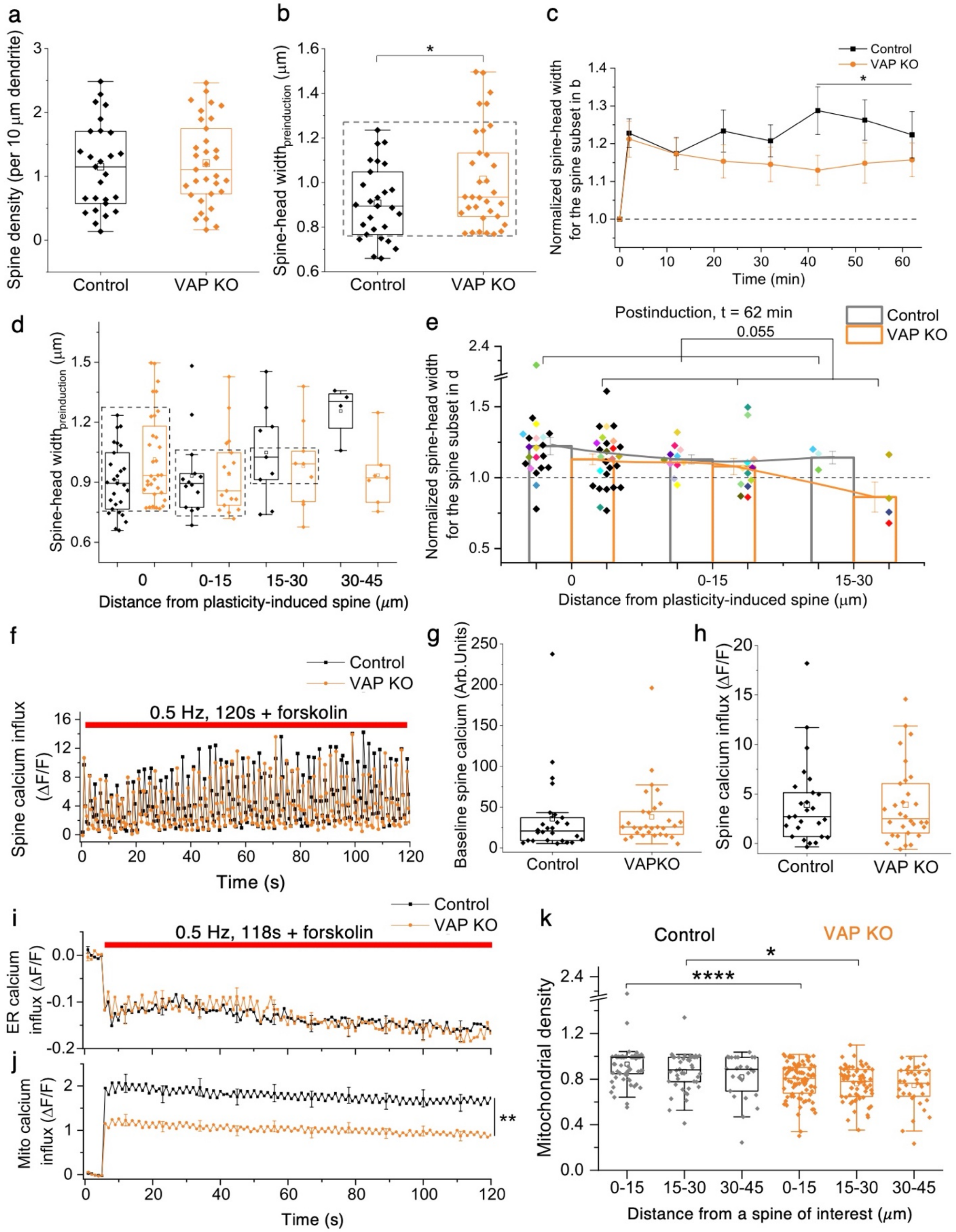

Figure 6. VAP deletion does not affect spine density, and spine and ER calcium, but affects mitochondrial calcium. a Spine density. n in dendrites, animals: 27, 11 (Control), 33, 7 (VAP

KO). Two sample t-Test, p: 0.74. **b** Spine-head width. Δspine $size_{increase}$ = 0.1 ± 0.07 μm; Control, 0.9 ± 0.03; VAP KO, 1.0 ± 0.04. n in spines, animals: 27, 11 (Control), 33, 7 (VAP KO). Two sample t-Test, p: 0.04. **c** Average time course of baseline-spine-head-width-matched spines from Control (black) and VAP KO (orange) in **b** (dotted box, spine size 0.76 – 1.3 μm). n in spines, animals: 21, 9 (Control), 28, 7 (VAP KO). One-way ANOVA, Tukey test, p: 0.025 (42-62 min). **d** Baseline spine-head width of plasticity-induced and adjacent spines within 0-15, 15-30, and 30-45 μm. For Control, n in spines, animals: 27, 11 (0 μm), 14, 8 (0-15 μm); 9, 5 (15-30 μm); 4, 2 (30-45 μm). For VAP KO, n: 32, 7 (0 μm); 17, 4 (0-15 μm); 9, 5 (15-30 μm); 7, 4 (30-45 μm). **e** Histogram of baseline-spine-head width-matched spines from Control (gray) and VAP KO (orange) in **d** (dotted boxes), 62 min post-plasticity induction. Individual points represent spines and are color-coded by neurons. n in spines, animals: 21, 9 (0 μm), 11, 6 (0-15 μm); 3, 3 (15-30 μm). For VAP KO, n: 27, 7 (0 μm); 12, 3 (0-15 μm); 4, 3 (15-30 μm). One-way ANOVA, Tukey test, p: 0.055 (0, 0-15, 15-30). Representative trace (**f**) of the average baseline spine calcium (**g**) and spine calcium influx (**h**) during synaptic plasticity. n in spines, animals: 26, 11 (Control), 30, 6 (VAP KO). Two sample t-Test, p: 0.85 (for **g**) and p: 1.0 (for **h**). **i,** Average ER calcium release. n in dendrites, animals: 84, 3 (Control), 76, 3 (VAP KO). Mann Whitney Test, p: 0.5. **j** Average mitochondrial calcium influx. n in dendrites, animals: 54, 3 (Control), 76, 3 (VAP KO). Mann-Whitney Test, p: 0.002. **k** Mitochondrial density within different distance ranges from spines ~110 μm from the cell body. n in dendrites, animals: 38, 2 (Control), 50, 2 (VAP KO). One-way ANOVA, Tukey test, p: 1.08 X 10^{-5} (0-15 μm), 0.019 (15-30 μm). Source Data files are provided.

Supplementary Tables

Table 1. 129 proteins found in the neuronal OMM proteome.

Column 1: UniProt ID of the OMM protome

Column 2: Gene names of the OMM proteome

Column 3: Protein names of the OMM proteome

Columns 4, 5, 6: Presence or absence of the OMM proteome in replicates R1, R2, and R3, respectively.

Column 7: GO annotation of the OMM proteome with the term "Mitochondria" revealed 26 proteins (20% of the OMM proteome), whereas the rest of the 103 proteins (80% of the OMM proteome) were not GO annotated as "Mitochondria" and were therefore OMM interacting proteins used for the screen.

Column 8: GO annotation of the OMM proteome with the term "Endoplasmic Reticulum" revealed 18 proteins (14% of the OMM proteome), of which 3 proteins – Dnm1l, Prkca, and Uba1 – are also GO annotated as "Mitochondria".

Column 9: Comparison of the neuronal OMM proteome with the published OMM+NES proteome and OMM exclusive proteome in HEK cells[2] revealed 69 overlapping proteins (53% of the neuronal OMM proteome).

Column 10: Actin (Actb) interactors in the OMM proteome revealed 18 proteins.

Column 11: Tubulin (Tuba1a) interactors in the OMM proteome revealed 6 proteins.

Column 12: Comparison of the OMM proteome with the most abundant soluble proteins in the neuropil proteome revealed only 22 (17%) overlapping proteins, confirming that the OMM proteome is not proteins merely bumping mitochondria by chance with no functional interaction.

Column 13: Comparison of the OMM proteome with all the soluble proteins in the neuropil proteome revealed only 31 (24%) overlapping proteins, confirming that APEX-OMM labeling is not too promiscuous in that it only results in soluble proteins.

UniProt ID	Gene Name	Protein Name	R1	R2	R3	GO Annotation "Mitochondria"	GO Annotation "Endoplasmic Reticulum"	OMM+NES proteome and OMM excl. proteome (Hung et al., eLife 2017)	Actin Interactors using BioGRID	Tubulin Interactors using BioGRID	Most abundant soluble proteins in the neuropil proteome	All soluble proteins in the neuropil proteome
A2VD09	abi1	Abl interactor 1	+		+			+			+	+
G3V9G4	acly	ATP-citrate synthase	+		+			+				+

F8WG67	acot7	Cytosolic acyl coenzyme A thioester hydrolase	+		+	+					+	+
A0A0G2JSM7	add1	Alpha-adducin		+	+			+				
P05065	aldoa	Fructose-bisphosphate aldolase A	+	+	+	+		+			+	+
A0A0G2K3Q6	aldoc	Fructose-bisphosphate aldolase C	+		+			+				
G3V6U3	alg2	Alpha-1,3/1,6-mannosyltransferase ALG2	+		+							
P07150	anxa1	Annexin A1	+		+	+						
Q3ZB97	ap2b1	AP complex subunit beta;AP-2 complex subunit beta	+	+	+			+	+			
D3ZPP2	arl8a	ADP-ribosylation factor-like GTPase 8A	+	+				+				
Q6PST4	atl1	Atlastin-1	+		+		+					
P07340	atp1b1	Sodium/potassium-transporting ATPase subunit beta-1		+	+							
P11507	atp2a2	Sarcoplasmic/endoplasmic reticulum calcium ATPase 2	+		+		+					
P11505	atp2b1	Plasma membrane calcium-transporting ATPase 1	+		+			+				
D4A8B3	atp2b2	Calcium-transporting ATPase;Plasma membrane calcium-	+		+		+					

		transporting ATPase 2										
Q2I6B2	atp6v0a1	V-type proton ATPase subunit a;V-type proton ATPase 116 kDa subunit a isoform 1	+	+	+			+				
D4A133	atp6v1a	H(+)-transporting two-sector ATPase	+	+	+			+	+			
P62815	atp6v1b2	V-type proton ATPase subunit B, brain isoform	+		+			+				
Q62717	cadps	Calcium-dependent secretion activator 1	+		+							
P11730	camk2g	Calcium/calmodulin-dependent protein kinase type II subunit gamma		+	+		+	+				
A0A0G2K1R5	camkv	CaM kinase-like vesicle-associated protein	+	+	+							
P97536	cand1	Cullin-associated NEDD8-dissociated protein 1	+		+			+			+	+
Q08163	cap1	Adenylyl cyclase-associated protein 1	+		+	+		+	+			
Q68FQ0	cct5	T-complex protein 1 subunit epsilon	+		+						+	+
Q3MHS9	cct6a	Chaperonin containing Tcp1, subunit 6A (Zeta 1)	+		+						+	+
A0A0G2JSM8	cdc42	Cell division	+	+	+			+	+	+		

		control protein 42 homolog										
Q5BJT9	ckmt1b	Creatine kinase U-type, mitochondrial	+		+							
Q99JD4	clasp2	CLIP-associating protein 2	+	+	+			+				
G3V624	coro1c	Coronin	+		+			+			+	+
D3ZGN2	cpne5	Copine-9	+		+							
D3ZPR0	cse1l	Chromosome segregation 1-like protein	+	+				+				
A0A0G2JT93	ctnnb1	Catenin beta-1	+		+			+				
A0A0G2K472	cyfip1	Cytoplasmic FMR1-interacting protein	+	+	+			+	+			
D3ZX82	cyfip2	Cytoplasmic FMR1 interacting protein 2 (Predicted)	+		+			+			+	+
A0A0G2K719	ddx3y	RNA helicase	+		+							
F1M3W5	dmxl2	Dmx-like 2	+		+			+				
Q5M9H7	dnaja2	DnaJ (Hsp40) homolog, subfamily A, member 2	+		+			+				
D4A0I5	dnajc6	DnaJ (Hsp40) homolog, subfamily C, member 6 (Predicted)	+		+							
O35303	dnm1l	Dynamin-1-like protein	+		+	+	+					
F1LNT0	dpysl4	Dihydropyrimidinase-related protein 4	+		+			+				
M0R9X8	dync1h1	Cytoplasmic dynein 1 heavy chain 1	+	+				+				

G3V7G0	dync1li1	Cytoplasmic dynein 1 light intermediate chain 1	+		+			+				+
P63170	dynll1	Dynein light chain 1, cytoplasmic;Dynein light chain 2, cytoplasmic	+		+	+		+	+	+		
Q68FR6	eef1g	Elongation factor 1-gamma	+		+		+	+			+	+
D4A9L5	epb41l1	Band 4.1-like protein 1	+		+							
P12785	fasn	Fatty acid synthase;[Acyl-carrier-protein] S-acetyltransferase;[Acyl-carrier-protein] S-malonyltransferase;3-oxoacyl-[acyl-carrier-protein] synthase;3-oxoacyl-[acyl-carrier-protein] reductase;3-hydroxyacyl-[acyl-carrier-protein] dehydratase;Enoyl-[acyl-carrier-protein] reductase; Oleoyl-[acyl-carrier-protein] hydrolase	+		+			+				
P85845	fscn1	Fascin	+		+			+			+	+
Q05683	gad2	Glutamate decarboxylase 2	+	+	+							

Q9JKB7	gda	Guanine deaminase	+		+	+					+	+
B4F774	gdap1l1	Ganglioside-induced differentiation-associated protein 1-like 1	+	+	+						+	+
Q9Z272	git1	ARF GTPase-activating protein GIT1	+		+	+		+				
P54313	gnb2	Guanine nucleotide-binding protein G(I)/G(S)/G(T) subunit beta-2;Guanine nucleotide-binding protein subunit beta-4	+		+							
A0A0G2K0Z7	gpd2	Glycerol-3-phosphate dehydrogenase, mitochondrial;Glycerol-3-phosphate dehydrogenase	+		+	+		+				
Q03555	gphn	Gephyrin;Molybdopterin adenylyltransferase;Molybdopterin molybdenumtransferase	+	+	+			+				
A0A1B0GWN8	gpm6a	Neuronal membrane glycoprotein M6-a	+		+							
M0R9A7	gria1	Glutamate receptor 1	+	+			+					
G3V914	gria2	Glutamate receptor 2	+		+		+					
A0A0G2JSH4	gsk3b	Glycogen synthase kinase-3 beta	+		+	+						

Q5D059	hnrnpk	Heterogeneous nuclear ribonucleoprotein K	+	+	+	+			+	+		+
P84076	hpca	Neuron-specific calcium-binding protein hippocalcin;Hippocalcin-like protein 1;Neurocalcin-delta	+	+	+							
P82995	hsp90aa1	Heat shock protein HSP 90-alpha	+	+	+	+		+				
A0A0G2JVH4	immt	MICOS complex subunit Mic60	+	+		+			+			
P10499	kcna1	Potassium voltage-gated channel subfamily A member 1;Potassium voltage-gated channel subfamily A member 2;Potassium voltage-gated channel subfamily A member 3	+		+		+					
A0A0G2K070	kif5c	Kinesin-like protein	+		+			+				
A0A140TAB3	klc1	Kinesin light chain 1	+		+							
D4A817	loc100912338	Histone H2B		+	+							
A0A0G2K6I0	loc691716	40S ribosomal protein S15a	+		+							
M9MMM9	macf1	Microtubule-actin cross-linking factor 1	+		+			+				

Q66HR2	mapre1	Microtubule-associated protein RP/EB family member 1	+		+			+				
A0A0G2JSR7	matr3	Matrin-3	+		+							+
O35095	ncdn	Neurochondrin	+		+			+				
F1LSM5	nckap1	Nck-associated protein 1	+		+			+	+			
A0A0G2K0B4	nedd4	E3 ubiquitin-protein ligase NEDD4	+		+			+				
A0JPJ7	ola1	Obg-like ATPase 1	+		+			+				+
Q2TA68	opa1	Dynamin-like 120 kDa protein, mitochondrial;Dynamin-like 120 kDa protein, form S1	+		+	+		+				
B2RYG6	otub1	Ubiquitin thioesterase OTUB1	+		+			+				
Q4V8F6	pcbp2	Pcbp2 protein	+		+						+	+
A0A140TAB1	pde4d	cAMP-specific 3,5-cyclic phosphodiesterase 4D	+	+								
C7C5T2	pfkp	ATP-dependent 6-phosphofructokinase;ATP-dependent 6-phosphofructokinase, platelet type	+	+	+						+	+
D3ZDU5	pfn2	Profilin;Profilin-2	+	+	+			+	+			
F1LSE6	ppfia3	Liprin-alpha-3	+		+			+				

Q80Z30	ppm1e	Protein phosphatase 1E	+	+	+	+					+	+
A0A0G2JYS8	ppp1cc	Serine/threonine-protein phosphatase;Serine/threonine-protein phosphatase PP1-gamma catalytic subunit;Serine/threonine-protein phosphatase PP1-beta catalytic subunit;Serine/threonine-protein phosphatase PP1-alpha catalytic subunit	+		+	+						
A0A0G2JYA4	ppp2ca	Serine/threonine-protein phosphatase 2A catalytic subunit alpha isoform;Serine/threonine-protein phosphatase 2A catalytic subunit beta isoform	+		+			+				
Q5XI34	ppp2r1a	Protein phosphatase 2 (Formerly 2A), regulatory subunit A (PR 65), alpha isoform, isoform CRA_a	+		+			+			+	+
P36876	ppp2r2a	Serine/threonine-protein	+		+			+			+	+

		phosphatase 2A 55 kDa regulatory subunit B alpha isoform;Serine/threonine-protein phosphatase 2A 55 kDa regulatory subunit B delta isoform										
P63329	ppp3ca	Serine/threonine-protein phosphatase 2B catalytic subunit alpha isoform	+		+	+		+				
A0A0G2K7T5	ppp3cb	Serine/threonine-protein phosphatase 2B catalytic subunit beta isoform	+		+							
A0A0H2UHV6	ppp3r1	Calcineurin subunit B type 1	+		+							
F1M2P8	prkca	Protein kinase C;Protein kinase C alpha type	+		+	+	+	+				
Q6NYB7	rab1a	Ras-related protein Rab-1A	+		+		+		+			
A1L1J8	rab5b	RAB5B, member RAS oncogene family	+		+							
A0A0G2K0X4	rac1	Ras-related C3 botulinum toxin substrate 1	+		+			+	+	+		
D3ZAS1	rgd1562399	40S ribosomal protein S2	+	+	+							

A1L1L6	rhot1	Mitochondrial Rho GTPase	+		+	+		+				
A0A0G2KBA1	rpl6	60S ribosomal protein L6	+		+		+		+			
Q6RJR6	rtn3	Reticulon-3	+		+		+					
F1LQN3	rtn4	Reticulon;Reticulon-4	+	+	+		+					
A0A0G2K6A9	rufy3	Protein RUFY3	+		+			+				
D3ZT07	sep5	Septin-5	+		+							
Q62634	slc17a7	Vesicular glutamate transporter 1	+	+								
A0A159KIL2	slc1a2	Amino acid transporter; Excitatory amino acid transporter 2	+		+							
A0A0G2K611	slc1a3	Amino acid transporter; Excitatory amino acid transporter 1	+		+	+						
F1LZW6	slc25a13	Solute carrier family 25 member 13	+		+	+						
P23978	slc6a1	Sodium- and chloride-dependent GABA transporter 1	+		+							
Q05140	snap91	Clathrin coat assembly protein AP180		+	+							
P37377	snca	Alpha-synuclein	+		+	+		+	+	+		
A0A0G2JSQ1	sncb	Beta-synuclein	+	+	+					+		
B2RZB7	snrpd1	Small nuclear ribonucleoprotein Sm D1	+		+							+
D4A208	srgap2	SLIT-ROBO Rho GTPase-	+		+			+	+			

		activating protein 2										
P61265	stx1b	Syntaxin-1B	+		+							
Q02563	sv2a	Synaptic vesicle glycoprotein 2A	+	+	+		+					
D4ABK1	syngr3	Synaptogyrin 3	+	+	+			+				
P07825	syp	Synaptophysin	+	+	+			+				
I6L9G6	tardbp	TAR DNA-binding protein 43	+		+							+
R9PXR4	tomm70a	Mitochondrial import receptor subunit TOM70	+		+	+		+				
G3V8D6	trim3	Tripartite motif-containing protein 3	+		+			+				+
P11232	txn	Thioredoxin	+		+							
Q5U300	uba1	Ubiquitin-like modifier-activating enzyme 1	+	+	+	+	+	+			+	+
D3ZC84	usp9x	Ubiquitin carboxyl-terminal hydrolase	+		+			+				
Q9Z270	vapa	Vesicle-associated membrane protein-associated protein A	+		+		+	+	+			
P46462	vcp	Transitional endoplasmic reticulum ATPase	+	+	+		+	+			+	+
B5DFC1	vps35	Vps35 protein	+		+	+		+				
Q56A29	vsnl1	Visinin-like protein 1	+		+							
Q5BJU7	wasf1	Wiskott-Aldrich syndrome protein family member 1	+		+	+		+			+	+
D3ZQ02	wdr37	WD repeat domain 37	+		+			+				
G3V9M3	wdr47	RCG28460	+	+	+			+				+

P35213	ywhab	14-3-3 protein beta/alpha; 14-3-3 protein beta/alpha, N-terminally processed	+		+			+	+		+	+
P68255	ywhaq	14-3-3 protein theta	+	+	+			+	+		+	+

Table 2. 18 actin interactors found in the OMM proteome

Column 1: UniProt ID of the actin (Actb) interactors in the OMM proteome

Column 2: Gene names of the actin interactors in the OMM proteome

Column 3: Protein names of the actin interactors in the OMM proteome and the 8 candidates investigated in this study (gray rows)

Columns 4, 5, 6: Presence or absence of the actin interactors in the OMM proteome in replicates R1, R2, and R3, respectively.

Column 7: GO annotation of the actin interactors in the OMM proteome with the term "Mitochondria" revealed 5 proteins, whereas the rest of the 13 proteins are not GO annotated as "Mitochondria".

Column 8: 18 Actin interactors found in the OMM.

Column 9: 5 Tubulin (Tuba1a) interactors that are also OMM-actin interactors.

UniProt ID	Gene Name	Protein Name	R 1	R 2	R 3	GO Annotation "Mitochondria"	Actin Interactor	Tubulin Interactor
A0A0G2JVH4	immt	MICOS complex subunit Mic60	+	+		+	+	
P37377	snca	Alpha-synuclein	+		+	+	+	+
D4A208	srgap2	SLIT-ROBO Rho GTPase-activating protein 2	+		+		+	
Q9Z270	vapa	Vesicle-associated membrane protein-associated protein A	+		+		+	
Q08163	cap1	Adenylyl cyclase-associated protein 1	+		+	+	+	
A0A0G2K472	cyfip1	Cytoplasmic FMR1-interacting protein	+	+	+		+	
F1LSM5	nckap1	Nck-associated protein 1	+		+		+	
D3ZDU5	pfn2	Profilin;Profilin-2	+	+	+		+	
D4A133	atp6v1a	H(+)-transporting two-sector ATPase	+	+	+		+	
A0A0G2KBA1	rpl6	60S ribosomal protein L6	+		+		+	
Q3ZB97	ap2b1	AP complex subunit beta;AP-2 complex subunit beta	+	+	+		+	
A0A0G2JSM8	cdc42	Cell division control protein 42 homolog	+	+	+		+	+
P63170	dynll1	Dynein light chain 1, cytoplasmic;Dynein light chain 2, cytoplasmic	+		+	+	+	+
Q5D059	hnrnpk	Heterogeneous nuclear ribonucleoprotein K	+	+	+	+	+	+

Q6NYB7	rab1a	Ras-related protein Rab-1A	+		+		+	
A0A0G2K0X4	rac1	Ras-related C3 botulinum toxin substrate 1	+		+		+	+
P35213	ywhab	14-3-3 protein beta/alpha;14-3-3 protein beta/alpha, N-terminally processed	+		+		+	
P68255	ywhaq	14-3-3 protein theta	+	+	+		+	

References

1 Chapman-Smith, A. & Cronan, J. E., Jr. Molecular biology of biotin attachment to proteins. *J Nutr* **129**, 477S-484S, doi:10.1093/jn/129.2.477S (1999).

2 Hung, V. *et al.* Proteomic mapping of cytosol-facing outer mitochondrial and ER membranes in living human cells by proximity biotinylation. *Elife* **6**, doi:10.7554/eLife.24463 (2017).
