# Supplementary material for: VAP spatially stabilizes dendritic mitochondria to locally support synaptic plasticity
Source: Nat Commun. 2024 Jan 4;15:205. doi: 10.1038/s41467-023-44233-8 (PMC10766606; doi:10.1038/s41467-023-44233-8)
Supplement: Supplementary file 3 — Reporting Summary [file 41467_2023_44233_MOESM3_ESM.pdf]

nature portfolio

Corresponding author(s): Vidhya Rangaraju

Last updated by author(s): Dec 4, 2023

Reporting Summary

Nature Portfolio wishes to improve the reproducibility of the work that we publish. This form provides structure for consistency and transparency in reporting. For further information on Nature Portfolio policies, see our Editorial Policies and the Editorial Policy Checklist.

Statistics

For all statistical analyses, confirm that the following items are present in the figure legend, table legend, main text, or Methods section.

n/a	Confirmed	
☐	☒	The exact sample size (*n*) for each experimental group/condition, given as a discrete number and unit of measurement
☐	☒	A statement on whether measurements were taken from distinct samples or whether the same sample was measured repeatedly
☐	☒	The statistical test(s) used AND whether they are one- or two-sided<br>*Only common tests should be described solely by name; describe more complex techniques in the Methods section.*
☒	☐	A description of all covariates tested
☐	☒	A description of any assumptions or corrections, such as tests of normality and adjustment for multiple comparisons
☐	☒	A full description of the statistical parameters including central tendency (e.g. means) or other basic estimates (e.g. regression coefficient) AND variation (e.g. standard deviation) or associated estimates of uncertainty (e.g. confidence intervals)
☐	☒	For null hypothesis testing, the test statistic (e.g. *F*, *t*, *r*) with confidence intervals, effect sizes, degrees of freedom and *P* value noted<br>*Give P values as exact values whenever suitable.*
☒	☐	For Bayesian analysis, information on the choice of priors and Markov chain Monte Carlo settings
☒	☐	For hierarchical and complex designs, identification of the appropriate level for tests and full reporting of outcomes
☐	☒	Estimates of effect sizes (e.g. Cohen's *d*, Pearson's *r*), indicating how they were calculated

Our web collection on statistics for biologists contains articles on many of the points above.

Software and code

Policy information about availability of computer code

Data collection	Dionex U3000 RSLCnano and Bruker NanoElute nano-HPLCs and Orbitrap Elite, Q Exactive Plus (Thermofisher) and Bruker Impact-II mass spectrometers were used for mass spectrometric analysis. The 3i imaging software and Slidebook 5.5 and 6 were used for image acquisition from the 3i spinning disc microscope. The Zeiss software Zen<br>2.3 SP1 FP2 (black) was used for image acquisition from the Zeiss Airyscan confocal microscope.
Data analysis	MaxQuant (version 1.6.0.1), Proteome Discoverer, Gene Ontology Resource, and BioGRID were used for mass spectrometry data analysis. A custom-written MATLAB (R2017b and R2019b) script was used to calculate the spine-head width. This script was previously published as cited in the manuscript. All other custom-written data analysis scripts are available at https://github.com/Rangaraju-Lab/Bapat et al 2023.git. The other image and data analysis software used were ImageJ 1.52S, IMARIS 8.4.1 and 9.5.1, Microsoft Excel 16.69.1, OriginPro 2022, and R 4.3.1.

For manuscripts utilizing custom algorithms or software that are central to the research but not yet described in published literature, software must be made available to editors and reviewers. We strongly encourage code deposition in a community repository (e.g. GitHub). See the Nature Portfolio guidelines for submitting code & software for further information.

Data

Policy information about availability of data

All manuscripts must include a data availability statement. This statement should provide the following information, where applicable:

- Accession codes, unique identifiers, or web links for publicly available datasets
- A description of any restrictions on data availability
- For clinical datasets or third party data, please ensure that the statement adheres to our policy

The mass spectrometry proteomics data generated in this study have been deposited in the ProteomeXchange Consortium via the PRIDE partner repository under accession code PXD047226 (https://www.ebi.ac.uk/pride/archive/projects/PXD047226). The raw imaging data will be provided by the corresponding author upon request. The source data generated in this study are provided in the Source Data file.

Research involving human participants, their data, or biological material

Policy information about studies with human participants or human data. See also policy information about sex, gender (identity/presentation), and sexual orientation and race, ethnicity and racism.

Reporting on sex and gender	n/a
Reporting on race, ethnicity, or other socially relevant groupings	n/a
Population characteristics	n/a
Recruitment	n/a
Ethics oversight	n/a

Note that full information on the approval of the study protocol must also be provided in the manuscript.

Field-specific reporting

Please select the one below that is the best fit for your research. If you are not sure, read the appropriate sections before making your selection.

☒ Life sciences ☐ Behavioural & social sciences ☐ Ecological, evolutionary & environmental sciences

For a reference copy of the document with all sections, see nature.com/documents/nr-reporting-summary-flat.pdf

Life sciences study design

All studies must disclose on these points even when the disclosure is negative.

Sample size	No statistical method was used to predetermine the sample size. Sample sizes were similar to or larger than those reported in the previous publications in the field and sufficient for our claims based on statistical significance.
Data exclusions	In Figure 6, spines were excluded from the analysis either when they did not respond to the uncaging stimulus, the spine showed swelling, disappeared during the period of imaging postinduction, or the Gaussian fit was poor. These criteria were preestablished. No further data points were excluded.
Replication	All experiments were repeated with neuronal culture dishes from at least two independent animals (except for Fig. 3d, 5i, Supplementary Fig. 3b, g, j). Each animal corresponds to one weekly batch of neuronal culture preparation. Within one animal experiment, at least 2 dishes were used per condition, and at least 4 transfected neurons were used for analysis. So, in cases where only 1 animal could be used, the individual neurons/dendrites/axons/spines data collected was sufficient to demonstrate reproducibility or for the claims based on statistical significance.
Randomization	Cell culture dishes were visually assessed for cell health and then randomly allocated to treatment and control groups.
Blinding	Investigators were not blind to conditions during image acquisition. However, most of the custom-written image or data analysis scripts processed the data from different conditions equally; therefore, the data analysis could be considered blinded.

Reporting for specific materials, systems and methods

We require information from authors about some types of materials, experimental systems and methods used in many studies. Here, indicate whether each material, system or method listed is relevant to your study. If you are not sure if a list item applies to your research, read the appropriate section before selecting a response.

Materials & experimental systems

n/a	Involved in the study
☐	☒ Antibodies
☒	☐ Eukaryotic cell lines
☒	☐ Palaeontology and archaeology
☐	☒ Animals and other organisms
☒	☐ Clinical data
☒	☐ Dual use research of concern
☒	☐ Plants

Methods

n/a	Involved in the study
☒	☐ ChIP-seq
☒	☐ Flow cytometry
☒	☐ MRI-based neuroimaging

Antibodies

Antibodies used

antibody-name (dilution, manufacturer, catalog number, clone if applicable):
anti-biotin rb (1:5000, Bethyl, A150109A)
anti-GFP chk (1:2000, AVES 1020)
anti-V5 ms (1:500, Invitrogen R960-25 (previously 460705), SV5-Pk1)
anti-FLAG ms (1:500, Sigma F3165, M2)
anti-Map2 rb (1:100, Abcam, ab32454)
anti-VAP A/B ms (1:100, NeuroMab 75496, N479/107)
anti-Vapa rb (1:100, Proteintech 152751AP)
anti-Vapb rb (1:100, Proteintech 144771AP)
anti-Snca ms (1:1000, BD biosciences 610787, 42/alpha-Synuclein)
anti-rb Alexa 405 (1:1000, Invitrogen A31556)
anti chk 608 Alexa 488 (1:1000, Invitrogen A11039)
anti-ms Alexa 546 (1:1000, Invitrogen A11030)
anti-ms Alexa 647 (1:1000, Invitrogen A21236)
anti-rb Alexa 488 (1:1000, Invitrogen A11008)

Validation

Each antibody was tested in immunocytochemistry in this and previous publications.
anti-Biotin: Recognizes biotin attached to proteins, peptides, oligonucleotides, or solid matrices. Validated for western blot (from manufacturer) and validated for immunochytochemistry in 'tom Dieck et al (2015) Direct visualization of identified and newly synthesized proteins in situ.
anti-GFP chk: Recognizes Green Fluorescent Protein (GFP). Validated for immunochytochemistry in 'Rangaraju et al (2019) Spatially stable mitochondrial compartments fuel local translation during plasticity'.
anti-V5 ms: Recognizes the V5 peptide. Validated for immunochytochemistry in 'Rhee et al (2013) Proteomic mapping of mitochondria in living cells via spatially-restricted enzymatic tagging'.
anti-FLAG ms: Recognizes the FLAG peptide. Validated for immunochytochemistry in 'Rhee et al (2013) Proteomic mapping of mitochondria in living cells via spatially-restricted enzymatic tagging'.
anti-Map2 rb: Reacts with rat (UniProt ID: P15146). Specific for MAP2; recognizes all four isoforms. Validated for immunocytochemistry in 'Rangaraju et al (2019) Spatially stable mitochondrial compartments fuel local translation during plasticity'.
anti-VAP A/B ms: Reacts with rat (UniProt ID: Q9WV55). Specific for VAP; recognizes both paralogs A and B. Validated for immunocytochemistry (from manufacturer)
anti-Vapa rb: Reacts with rat (UniProt ID: Q9WV55). Specific for the Vapa paralog. Validated for immunocytochemistry (from manufacturer)
anti-Vapb rb: Reacts with rat (UniProt ID: Q9Z269). Specific for the Vapb paralog. Validated for immunocytochemistry (from manufacturer)
anti-Snca ms: Reacts with rat (UniProt ID: P37377). Specific for the alpha synuclein. Validated for immunocytochemistry (from manufacturer)

Animals and other research organisms

Policy information about studies involving animals; ARRIVE guidelines recommended for reporting animal research, and Sex and Gender in Research

Laboratory animals	Primary hippocampal neuronal cultures were prepared as described in Methods from Sprague Dawley rats (age: PO-P1, mixed gender; Charles River).
Wild animals	No wild animals were used.
Reporting on sex	The sex of pups from which neurons were isolated was not determined.
Field-collected samples	No field-collected samples were used.
Ethics oversight	All experiments were performed according to the Max Planck Florida Institute for Neuroscience IACUC regulations (protocol number: 22-005).

Note that full information on the approval of the study protocol must also be provided in the manuscript.

Plants

Seed stocks	n/a
Novel plant genotypes	n/a
Authentication	n/a
